# Quality of care and 30 day mortality among patients with hip fractures: a nationwide cohort study

**DOI:** 10.1186/1472-6963-9-186

**Published:** 2009-10-12

**Authors:** Katrine A Nielsen, Niels C Jensen, Claus M Jensen, Marianne Thomsen, Lars Pedersen, Søren P Johnsen, Annette Ingeman, Paul D Bartels, Reimar W Thomsen

**Affiliations:** 1 Danish Institute for Quality and Accreditation in Healthcare, DK-8200 Aarhus N, Denmark; 2 Department of Orthopaedic Surgery, Aarhus University Hospital, DK-8000 Aarhus C, Denmark; 3 Department of Orthopaedic Surgery, Herlev University Hospital, DK-2730 Herlev, Denmark; 4 Department of Physio- and Occupational Therapy, Sydvestjysk Sygehus, DK-6700 Esbjerg, Denmark; 5 Department of Clinical Epidemiology, Aarhus University Hospital, DK-9000 Aalborg, Denmark; 6 The Coordinating Secretariat (DNIP), Regionshuset, DK-8200 Aarhus N, Denmark

## Abstract

**Background:**

We examined the association between quality of care and 30 day mortality in a nationwide cohort of patients hospitalized with hip fracture.

**Methods:**

We used data from The Danish National Indicator Project, a quality improvement initiative with participation of more than 90% of Danish hospital departments caring for patients with hip fracture between August 16, 2005 and August 15, 2006. Quality of care was measured in terms of meeting five specific criteria: early assessment of the patient's nutritional risk, systematic pain assessment during mobilization, assessment of Activities of Daily Living (ADL) before the fracture, assessment of ADL before discharge, and initiation of treatment to prevent future osteoporotic fractures. The association between meeting each of the quality of care criteria for the patient and 30 day mortality was examined using logistic regression to adjust for potential confounders.

**Results:**

6,266 patients hospitalized with an incident episode of hip fracture were included in the study. For four of the five quality of care criteria, patients who met the criterion had substantially lower 30 day mortality after hip fracture. The adjusted mortality odds ratios (ORs) ranged from 0.42 (95% CI, 0.30 to 0.58) for assessment of ADL before discharge (excluding deaths during hospitalization) to 0.72 (95% CI, 0.52 to 1.00) for systematic pain assessment. We found an inverse dose-response relationship between the number of quality of care criteria met and 30 day mortality; the lowest mortality was found among patients for whom all five quality of care criteria were met, as compared with patients for whom no quality of care criteria were met: adjusted mortality OR 0.18 (95% CI, 0.09 to 0.36).

**Conclusion:**

Higher quality of care during hospitalization with hip fracture was associated with lowered 30 day mortality.

## Background

Hip fracture is a major clinical and public health problem and the one condition associated with the largest use of bed days in hospitals in the Western World [1,2]. In Denmark there are approximately 9,500 annual hospital admissions with hip fractures, corresponding to 1 episode per 100 persons aged over 65 years per year [3]. Mortality within 30 days after hospitalization with a hip fracture ranges from 6% to 13% [1,4-10]. Moreover cohort studies have demonstrated excess mortality in patients with hip fracture compared with sex- and age-matched controls for up to ten years after the fracture [10-13].

Several studies have used a before-after design to examine the effect of implementation of treatment guidelines or care pathways for hip fracture patients with inconsistent results [14-21]. To our knowledge, only one cohort study from the U.S. examined the effect of meeting specific processes of treatment and care on the prognosis for individual patients. This study found that meeting a scale of processes of care for patients with hip fracture was associated with improved functional outcomes after two months but not after six months and not with mortality [22].

A recent nationwide Danish study among patients with stroke found that meeting six individual quality of care criteria was associated with lowered 30 and 90 day mortality [23]. As prevalence of hip fracture increases in the ageing Western populations [10,24], prevention of hip fracture-related deaths has significant clinical and public health importance. We examined the association between quality of treatment and care defined as meeting five quality of care criteria and 30 day mortality among patients with hip fracture in a nationwide population-based cohort study.

## Methods

### The Danish National Indicator Project (DNIP)

The Danish National Health Service provides tax-supported health care for all inhabitants of Denmark, including free access to hospital care. DNIP is a nationwide initiative to document, monitor and improve the quality of treatment and care provided by the Danish health care system. The project started in 2000 and focuses on developing and implementing evidence-based indicators related to health care structure, process, and outcomes [23,25]. Hip fracture is one of eight specific diseases monitored in the DNIP by clinical indicators and quality standards. All patients ≥ 65 years admitted to Danish hospitals with a hip fracture, *i.e*., medial, pertrochanteric or subtrochanteric femur fracture are eligible for inclusion in the DNIP database. All Danish hospital departments (except from hospitals in the Copenhagen Hospital Corporation) caring for patients with hip fracture participated in the project during the study period. Thus a total of 28 hospitals (32 departments, more than 90% of all hospital departments caring for patients with hip fractures) reported data on hip fracture to DNIP in 2006. Each participating hospital department in the DNIP receives on a continuous basis their own results on the proportion of their patients meeting a number of quality of care criteria (see below). In the DNIP database, individual-level data for hip fracture patients from all hospitals are stored, including prospectively collected patient characteristics and the type and number of quality of care criteria each patient met during hospital stay.

### Study population

We identified all patients with a hip fracture in the DNIP database who had a discharge date between August 16, 2005 and August 15, 2006 (N = 6,456). We excluded patients with multiple hip fractures during this study period to include only the first recorded hip fracture (n = 6,336). We excluded a few patients (n = 4) whose admission date was erroneously recorded to be later than the hip fracture operation or discharge date, and patients with more than one year time span between hospital admission and discharge (n = 2). Finally, we excluded patients with missing data for all five quality of care criteria (n = 64). Our study cohort thus comprised 6,266 patients with incident hip fracture.

### Quality of care criteria

A Danish national expert panel including physicians, nurses, physiotherapists, and occupational therapists identified five quality of care criteria to be measured during hospitalization with a hip fracture. The choice of criteria was based on a systematic search of the scientific literature and considerations regarding the feasibility of collecting the required data in routine clinical settings [25]. The five specific criteria are: early assessment (within 2 days after admission) of the patient's nutritional risk, systematic pain assessment during mobilization of the patient, assessment of Activities of Daily Living (ADL) before the fracture, assessment of ADL before discharge, and initiation of treatment to prevent future osteoporotic fractures. Assessment of nutritional risk was done according to updated nationwide guidelines. Systematic pain assessment had to be done using a pain scale with documented validity. Assessment of ADL before the fracture and again before discharge had to be done using a specific test to assess functional disability. Initiation of treatment to prevent future osteoporotic fractures was defined as ordination of any anti-osteoporotic medications or hip protectors. For two criteria, systematic pain assessment and prevention of future osteoporotic fractures, it was possible to classify patients as non-eligible: for instance if the patient had dementia or hemiplegia and therefore could not state their level of pain during mobilization, or if the patient already received anti-osteoporotic medication or had contraindications for treatment.

During hospitalization, hospital staff collected data on if the five quality of care criteria were met, as well as demographic characteristics and a range of prognostic covariates for each patient using a standardized form. After hospital discharge the computerized patient data were entered into the nationwide central DNIP database by the hospital staff.

### Mortality

Information on mortality during the study's follow-up period was obtained through linkage with the Danish Civil Registration System, which since 1968 has maintained electronic records of changes in vital status and migration for the entire Danish population [26].

### Patient characteristics

At the time of hospital admission, data was collected on the following patient characteristics: age, gender, living situation (living together with another adult, living alone in one's own home, or other including living in a nursing home or other institution), alcohol intake (≤ 14/21 or >14/21 drinks per week for women/men, respectively), and smoking habits (current, former, never), respectively.

During the hospitalization, data was collected on the following disease-related covariates: type of fracture (medial, pertrochanteric, subtrochanteric), fracture displacement (displaced, undisplaced), delay before surgery (< 23 hours, 24-47 hours, 48-71 hours, and more than 72 hours), the American Society of Anaesthesiologists' (ASA) classification score before surgery (healthy, mild systemic disease, severe but not incapacitating systemic disease, incapacitating and life-threatening systemic disease, or moribund patient not expected to survive for 24 h), and type of surgery (osteosynthesis, hemi alloplastic, total alloplastic, other operation), respectively.

To adjust for the individual burden of comorbidity in our mortality analyses, we computed Charlson comorbidity index scores for all patients with hip fracture using records of all hospital discharge diagnoses made prior to the admission date in the Danish hospital discharge registry. This registry was established in 1977 and collects data on all hospitalizations, including dates of admission and discharge, surgical procedure(s) performed, and up to 20 discharge diagnoses assigned by the treating physician and coded according to the International Classification of Diseases (8^th ^revision (ICD-8) until the end of 1993, and 10^th ^revision (ICD-10) thereafter).

The Charlson comorbidity [27] index covers 19 major disease categories, weighted according to their prognostic impact on patient survival, and has been validated for use with discharge registry data in the ICD databases for prediction of mortality [28]. We defined three levels of comorbidity for each patient, based on the complete hospital discharge history since 1994, as follows: 'low' for patients with no recorded underlying diseases included in the Charlson index; 'medium' (score 1-2); and 'high' (score >2).

The study was approved by The Danish Data Protection Agency (record no. 2007-41-0073).

### Statistical analysis

We used logistic regression to compute odds ratios (ORs) with 95% confidence intervals (CIs) for mortality at day 30 after hip fracture according to type and number of quality of care criteria met, adjusting for age, sex, the patient's living situation, and the Charlson comorbidity index score. In the second model we additionally adjusted for acute disease-related variables, i.e., type of fracture, fracture displacement status, ASA classification score, delay before surgery, and type of surgery, respectively. Because alcohol and smoking data was missing for 28.3% and 29.1% of the patients, respectively, the full model was run both with and without inclusion of alcohol and smoking data.

In all adjusted analysis, we used a random effect model to correct for possible clustering by hospital, because other and unmeasured quality of care characteristics at the health care provider level might be associated with patient mortality [29].

We first examined the association between each individual quality of care criterion met and 30 day mortality. Patients were excluded from the analysis if there was missing data if the specific quality of care criterion was met (ranging from 0.4% of patients for assessment of nutritional risk or ADL before the fracture to 15.4% for systematic pain assessment) or if the criterion was found non-eligible to the patient (i.e. if the patient had contraindications). Thus, the number of patients included in the analyses of the specific quality of care criteria differed. We ran analyses both for the entire patient cohort with use of separate categories for missing data on prognostic characteristics, and for a cohort of patients who had complete data on all prognostic characteristics. We also examined whether the quality of care criteria were independently associated with 30 day mortality by including all of the criteria into one model and thereby mutually adjusting them. This analysis was only possible to do among patients who were eligible for all quality of care criteria.

Secondly, we examined the association between the number of quality of care criteria met and 30 day mortality. For this analysis, we only included patients found eligible for all quality of care criteria. In an alternative model, we examined the association between the proportion (0-20%, 21-40%, 41-60%, 61-80%, and 81-100%) of all *relevant *quality of care criteria met and mortality within 30 days, also including patients for whom one or two criteria were found non-eligible.

Thirdly, we did a number of subanalyses to test the robustness of our findings. To reduce confounding by contraindication in patients near end-of-life we reran analyses with exclusion of patients who died during hospitalization. We also ran analyses for 30 day post-discharge mortality among all patients discharged alive, and for 180 days of follow-up both post-admission and post-discharge. All statistical analyses were performed with STATA™ software (version 8.2).

## Results

Table 1 shows demographic and clinical characteristics of the 6,266 patients hospitalized with a hip fracture. The median age was 83, and 74% of the study population were women. Nearly one half of the patients were living alone.

**Table 1:** Descriptive characteristics of 6266 patients with hip fracture registered in the Danish National Indicator Project.

**Characteristic**	**N (%)**
Age, years*	83.2 (65.0-107.8)
**Gender:**	
- Women	4626 (73.8%)
- Men	1640 (26.2%)
**Living situation:**	
- Living with someone	1636 (26.1%)
- Living alone	3176 (50.7%)
- Other	1025 (16.4%)
- Missing data	429 (6.9%)
**Alcohol intake†:**	
- < 14/21 drinks per week	4305 (68.7%)
**- **> 14/21 drinks per week	186 (2.9%)
- Missing data	1775 (28.3%)
**Smoking habits:**	
**- **Current smoker	1329 (21.2%)
- Former smoker	980 (15.6%)
**- **Never smoked	2133 (34.0%)
**- **Missing data	1824 (29.1%)
**Type of fracture:**	
**- **Medial	3187 (50.9%)
**- **Pertrochanteric	2564 (40.9%)
**- **Subtrochanteric	392 (6.3%)
**- **Unknown	123 (2,0%)
**Fracture displacement:**	
**- **Displaced	4598 (73.4%)
- Undisplaced	907 (14.5%)
- Unknown	761 (12.1%)
Delay before surgery, days*:	1 (0-124)
**Type of surgery:**	
- Osteosynthesis	4493 (71.7%)
**- **Hemi alloplastic	1320 (21.1%)
- Total alloplastic	218 (3.5%)
- Other operation	28 (0.5%)
- Unknown	207 (3.3%)
**ASA-score:**	
- Class 1: Healthy	584 (9.3%)
- Class 2: Mild systemic disease	2916 (46.5%)
- Class 3: Severe systemic disease not incapacitating	2042 (32.6%)
- Class 4: Incapacitating life-threatening systemic disease	324 (5.2%)
- Class 5: Moribund patient not expected to survive for 24 h	9 (0.1%)
Missing data	391 (6.2%)
**Charlson comorbidity index score:**	
- 0 points	2442 (39.0%)
- 1-2 points	2584 (41.2%)
- 3+ points	1240 (19.8%)

August 16, 2005 - August 15, 2006.

* Age and operation delay are stated as median (minimum-maximum)

† Drinks per week for women/men, respectively

The quality of care criteria were met for 30% of patients with respect to systematic pain assessment, for 35% of the patients concerning assessment of nutrition risk, for 39% of the patients concerning initiation of treatment to prevent future osteoporotic fractures, for 66% of the patients with respect to assessment of ADL before discharge, and for 69% concerning assessment of ADL before the hip fracture. The overall 30 day mortality in our cohort was 10.3%.

Table 2 presents crude and adjusted odds ratios (ORs) for death within 30 days according to quality of care criteria met. For four of the five quality of care criteria, patients who met the criterion had substantially lower 30 day mortality. Adjusted ORs for death in the full model ranged from 0.28 (95% CI 0.21 to 0.37) for assessment of ADL before discharge to 0.72 (95% CI 0.52 to 1.00) for systematic pain assessment during mobilization. For assessment of the patient's nutritional risk 30 day mortality was not reduced. The mortality estimates were not materially influenced by the statistical model used (i.e., excluding patients with missing data on prognostic characteristics or separate categories for missing data these factors). When we included all quality of care criteria in the same regression model for their mutual adjustment, systematic pain assessment (adjusted OR 1.25, 95% CI 0.83 to 1.88) and assessment of ADL before the fracture (adjusted OR 1.12, 95% CI 0.69 to 1.82) lost their independent association with reduced mortality. Mutual adjustment had only a minor impact on the other three criteria's association with mortality, with the lowest adjusted ORs found for assessment of ADL before discharge (OR 0.21, 95% CI 0.15 to 0.30) and prevention of future osteoporotic fractures (OR 0.57, 95% CI 0.38 to 0.85).

**Table 2:** Relationship between quality of care criteria and 30 day mortality after admission with hip fracture.

**Criteria met**	**Proportion of patients who died** **N (%)**	**Crude OR** **(95% CI)**	Adjusted OR* (95% CI) **Model 1**	**Adjusted OR†** **(95% CI)** **Model 2**
**Assessment of nutritional risk within 2 days after admission**				
Yes	199/2204 (9.3)	0.82 (0.69 to 0.98)	0.93 (0.75 to 1.14)	0.98 (0.77 to 1.19)
No	434/4036 (10.8)	1.00 (reference)	1.00 (reference)	1.00 (reference)
**Systematic pain assessment during mobilization**				
Yes	107/1886 (5.7)	0.58 (0.46 to 0.74)	0.66 (0.47 to 0.92)	0.72 (0.52 to 1.00)
No	228/2440 (9.3)	1.00 (reference)	1.00 (reference)	1.00 (reference)
**Assessment of ADL before the fracture**				
Yes	331/4360 (7.6)	0.43 (0.36 to 0.51)	0.47 (0.33 to 0.66)	0.54 (0.39 to 0.76)
No	302/1881 (16.0)	1.00 (reference)	1.00 (reference)	1.00 (reference)
**Assessment of ADL before discharge**				
Yes	227/4122 (5.5)	0.25 (0.21 to 0.30)	0.27 (0.20 to 0.36)	0.28 (0.21 to 0.37)
No	400/2107 (19.0)	1.00 (reference)	1.00 (reference)	1.00 (reference)
**Initiation of treatment to prevent future osteoporotic fractures**				
Yes	157/2434 (6.5)	0.58 (0.47 to 0.71)	0.66 (0.49 to 0.88)	0.64 (0.48 to 0.85)
No	293/2742 (10.7)	1.00 (reference)	1.00 (reference)	1.00 (reference)

* Adjusted for age, gender, comorbidity and living situation.

**† **Adjusted for age, gender, comorbidity, living situation, type of fracture, fracture displacement status, ASA-score, delay until surgery and type of surgery.

Table 3 shows adjusted ORs for 30 day mortality by number of quality of care criteria met among patients who were eligible for all the quality of care criteria. We found an inverse dose-response relationship between the number of quality of care criteria met and 30 day mortality. The lowest 30 day mortality was found among patients for whom all five quality of care criteria were met, compared to patients for whom no quality of care criteria were met: adjusted mortality OR 0.18 (95% CI 0.09 to 0.36) (Table 3). A similar trend was found when analyses were done for the proportion of relevant quality of care criteria met: Using 0-20% of criteria met as the reference category, adjusted ORs were 1.00 (0.63-1.57), 0.45 (0.27-0.73, 0.46 (0.28-0.75) and 0.28 (0.18-0.44) for 21-40%, 41-60%, 61-80%, and 81-100% of criteria met, respectively.

**Table 3:** Relationship between number of quality of care criteria met and 30 day mortality.

**Number of quality of care criteria met**	**Proportion of patients who died** **N (%)**	**Crude OR** **(95% CI)**	Adjusted OR* (95% CI) **Model 1**	**Adjusted OR†** **(95% CI)** **Model 2**
0	73/370 (19.7)	1.00 (reference)	1.00 (reference)	1.00 (reference)
1	36/362 (9.9)	0.45 (0.29 to 0.69)	0.55 (0.33 to 0.90)	0.68 (0.41 to 1.12)
2	54/811 (6.7)	0.29 (0,20 to 0.42)	0.31 (0.19 to 0.50)	0.34 (0.20 to 0.57)
3	40/812 (4.9)	0.21 (0.14 to 0.32)	0.25 (0.15 to 0.41)	0.30 (0.18 to 0.52)
4	22/819 (2.7)	0.11 (0.07 to 0.18)	0.13 (0.07 to 0.24)	0.17 (0.10 to 0.29)
5	16/510 (3.1)	0.13 (0.08 to 0.23)	0.17 (0.08 to 0.34)	0.18 (0.09 to 0.36)

Risk estimates calculated among hip fracture patients eligible for all quality of care criteria.

* Adjusted for age, gender, comorbidity and living situation.

**† **Adjusted for age, gender, comorbidity, living situation, type of fracture, fracture displacement status, ASA-score, delay until surgery and type of surgery.

Table 4 shows adjusted mortality ORs for fulfillment of each quality of care criteria according to different sensitivity analyses and follow-up periods up till 180 days after discharge. When we included only the 5,948 patients who were alive at discharge in the analyses (318 patients who died during hospitalization excluded) the association between quality of care and 30 day post-admission mortality weakened but remained statistically significant (Table 4). When we analyzed 30 day post-discharge mortality among all patients discharged alive we found further weakened associations, but for four quality of care criteria, patients who met the criterion still had lower mortality with adjusted ORs between 0.55 and 0.80. Similar mortality associations were found for 180 days of follow-up post-admission or post-discharge (Table 4). Finally, when we reran 30-day mortality analyses separately for the three largest hospitals caring for hip fracture patients in Denmark, we found associations between meeting each of the quality of care criteria and lower mortality that were qualitatively similar to the associations found on the nationwide level.

**Table 4:** Relationship between quality of care criteria and mortality in different sensitivity analyses.

Adjusted * mortality OR (95% CI) for fulfilment of the quality of care criteria	**Assessment of nutritional risk within 2 days after admission**	**Systematic pain assessment during mobilization**	**Assessment of ADL before the fracture**	**Assessment of ADL before discharge**	**Initiation of treatment to prevent future osteoporotic fractures**
**30-day post-admission mortality**	0.98 (0.77 to 1.19)	0.72 (0.52 to 1.00)	0.54 (0.39 to 0.76)	0.28 (0.21 to 0.37)	0.64 (0.48 to 0.85)
**30-day post-admission mortality excluding patients who died during hospitalization**	1.01 (0.76 to 1.34)	0.73 (0.54 to 0.99)	0.65 (0.49 to 0.87)	0.42 (0.30 to 0.58)	0.73 (0.54 to 0.99)
**30-day post-discharge mortality**	0.98 (0.78 to 1.22)	0.80 (0.64 to 1.02)	0.71 (0.53 to 0.94)	0.55 (0.41 to 0.73)	0.80 (0.63 to 1.01)
**180-day post-admission mortality**	0.92 (0.78 to 1.10)	0.79 (0.63 to 0.99)	0.67 (0.53 to 0.85)	0.46 (0.37 to 0.58)	0.75 (0.61 to 0.91)
**180-day post-discharge mortality**	0.93 (0.76 to 1.12)	0.79 (0.65 to 0.96)	0.76 (0.61 to 0.94)	0.61 (0.49 to 0.76)	0.81 (0.67 to 0.97)
**30-day post-admission mortality, largest hospital**	0.38 (0.14 to 1.14)	0.22 (0.02 to 2.27)	0.50 (0.18 to 1.37)	0.50 (0.18 to 1.44)	0.40 (0.14 to 1.14)
**30-day post-admission mortality, second largest hospital**	0.69 (0.23 to 2.05)	NA†	0.03 (0.01 to 0.15)	0.03 (0.01 to 0.15)	NA†
**30-day post-admission mortality, third largest hospital**	0.97 (0.51 to 1.83)	0.78 (0.33 to 1.81)	0.60 (0.19 to 1.84)	0.18 (0.09 to 0.37)	0.72 (0.36 to 1.43)

* Adjusted for age, gender, comorbidity, living situation, type of fracture, fracture displacement status, ASA-score, delay until surgery and type of surgery. For post-discharge mortality ORs, also adjusted for length of hospital stay.

† No deaths among patients with systematic pain assessment or initiation of treatment to prevent future osteoporotic fractures.

## Discussion

We found that a higher quality of care offered to patients with a hip fracture, defined as meeting specific quality of care criteria, was associated with substantially lower 30 day mortality for most criteria met. The association remained robust after adjusting for a range of possible confounding factors and in several sensitivity analyses done. We also found that the association between the number of quality of care criteria met and 30 day mortality appeared to follow an inverse dose-response pattern.

The strengths of our study are its size, the nationwide population-based design, the complete follow-up for ascertainment of survival status, and our ability to adjust for a wide range of potential confounders through access to independent medical databases providing a complete medical history.

Study limitations include the use of possibly inaccurate data collected during routine clinical work in a large number of settings. However, participation in DNIP is mandatory for all departments in Denmark treating patients a hip fracture and extensive efforts are made to ensure the validity of DNIP data [25]. Thus a structured audit process is carried out regularly on a national, regional, and local basis to assess critically the quality of the dataset and results. To ensure completeness of patient registration in DNIP, its enrollees are compared with local hospital discharge registries.

Patients who were classified as non-eligible or with missing data for the respective quality of care criteria examined had to be excluded in our study. Moreover, some patients had missing data for prognostic characteristics, but in general the association found when excluding those with missing data did not differ considerably from that based on separate prognostic categories for missing data, which indicates that the results are robust and reliable.

Importantly, our results may have been influenced by confounding by contraindication, e.g., the staff may have been less likely to offer early and appropriate care for frail patients near end-of-life with poor baseline survival chance. However, we were able to adjust for a wide range of individual prognostic predictors, including both the patients' entire medical history via the Charlson index score and the surgeon's or anesthesiologist's acute evaluation of the patient's baseline risk of death via the ASA score. Furthermore, the staff had the possibility to consider patients ineligible for several of the care processes, for example, if a patient was found too weak to participate in mobilization or systematic pain assessment. These patients were subsequently excluded from our analysis of the quality criterion. On this basis, it does not appear very likely that the staff would deliberately withhold care to patients with a severe prognosis whom they had considered eligible to the specific care processes. Even excluding all patients who died during hospitalization did not eliminate the association between quality of care and 30 day post-admission mortality. Slightly weaker but consistent associations were also observed for post-discharge mortality follow-up at both 30 and 180 days. Thus, we find it unlikely that unmeasured or unknown confounders could explain mortality decreases of the magnitude observed.

There are also some limitations with regard to selection and definition of the quality of care criteria in the DNIP. The criteria were deliberately selected to primarily reflect the quality of care in the early phase of a hip fracture as in-hospital care is the focus of DNIP. A range of other processes of care, including long-term rehabilitation which is typically undertaken by other parts of the health care system including the municipalities, are also highly relevant for patients with hip fracture and the association between the quality of such processes and mortality remains to be clarified.

Most of the quality of care criteria used in DNIP hip fracture are proxy measures for processes believed to influence the prognosis and mortality among patients with hip fracture. For example, assessment of Activities of Daily Living (ADL) before the fracture and again before discharge cannot *per se *reduce mortality; however, assessment may promote adequate mobilization and rehabilitation, eventually leading to an improved prognosis. Poor nutritional status is associated with increased length of stay [30] and mortality [31] following hip fracture, and supplementary food intake may decrease mortality [31]. Assessment of the patient's nutritional risk forms the basis for nutritional therapy. There is similarly good evidence that undertreated postoperative pain contributes to increased length of stay and enhanced recovery following hip fracture [32-34]. Systematic pain assessment may promote improved pain control [34]. Finally, there is increased risk of early subsequent fracture after a first hip fracture [35]. Anti-osteoporotic treatment such as calcium and vitamin D supplementation may decrease future hip fracture risk [36] and be associated with other beneficial outcomes such as decreased risk of infection [37].

Finally, we focused on mortality as the outcome in this analysis. Despite its obvious importance, mortality is certainly not the only relevant end-point for patients with hip fracture. Examination of association between quality of care and other end-points, *e.g*., functional level after discharge would of course also be highly relevant. Unfortunately, data on functional level were not available in our study.

It is difficult to compare our study with previous research, because to our knowledge no prior studies have examined the association between meeting the specific processes of care used in our study and 30 day mortality. Most previous before-and-after studies examined the effects of implementation of specific sets of guidelines or care pathways rather than the effect of meeting specific individual processes of treatment and care. The only study [22] that examined the effect of meeting processes of treatment and care in hip fracture found that individual processes of treatment and care were not associated with functional outcomes or mortality after adjustment for potential confounding variables. However, the study found that meeting the whole scale of processes of care was associated with fewer readmissions and better locomotion, self-care, and transferring (from for example the bed or toilet) at two months but not at six months. Meeting the whole scale of processes of care was not associated with mortality at 6 months (Mortality Hazard Ratio = 0.95, P-value > 0.5), whereas the authors did not examine mortality after 30 days as in our study.

## Conclusion

In conclusion, our large nationwide population-based cohort study found that high quality of treatment and care offered to patients with a hip fracture, defined by meeting specific quality of care criteria, was associated with substantially lowered 30 day mortality. The association between the number of quality of care criteria met and 30 day mortality appeared to follow an inverse dose-response pattern. These findings indicate that it is possible to link process and outcome and thereby provide direct support for implementation of evidence-based treatment and care to patients with hip fracture.

## Competing interests

The authors declare that they have no competing interests.

## Authors' contributions

KAN, NCJ, SPJ, PDB and RWT conceived and designed the study. KAN was the principal investigator and lead author in the analysis of the data and wrote the first draft of the manuscript. LP participated in the study design and provided statistical suggestions. All authors participated in the interpretation of the findings. All authors took part in reviewing and editing the manuscript, and approved the final version to be published.

## Pre-publication history

The pre-publication history for this paper can be accessed here:


